# Sacrococcygeal Yolk Sac Tumor: An Uncommon Site

**Published:** 2012-09-01

**Authors:** Fatma Khanchel-Lakhoua, Wafa Koubâa-Mahjoub, Raja Jouini, Meriem Bel Haj Salah, Néjib Kaabar, Achraf Chadli-Debbiche

**Affiliations:** Department of Pathology, Habib Thameur Hospital, Tunis, Tunisia; Department of Pathology, Habib Thameur Hospital, Tunis, Tunisia; Department of Pathology, Habib Thameur Hospital, Tunis, Tunisia; Department of Pathology, Habib Thameur Hospital, Tunis, Tunisia; Dept. of Pediatric Surgery, Habib Thameur Hospital, Tunis, Tunisia; Department of Pathology, Habib Thameur Hospital, Tunis, Tunisia

**Keywords:** Segmental dilatation, Colon, Colorraphy

## Abstract

A 30-month-old male infant presented with sacrococcygeal and pre-sacral mass. Ultrasound (US) abdomen revealed a huge pre-sacral mass with irregular margins extending into the pelvis, pushing the rectum antero-laterally. CT scan and MRI confirmed the US findings. Serum alpha fetoprotein level was abnormally elevated. Histopathological examination of surgical-specimen suggested sacrococcygeal yolk sac tumor (YST).

## INTRODUCTION

The majority of sacrococcygeal tumors are benign, teratomas [1]. These tumors however have the potential for malignant degeneration. Malignancy is usually limited to a single element, a yolk sac tumor also known as endodermal sinus tumor (EST) [2]. This tumor may less commonly be present in "pure" form. Malignant germ cell tumors account for 3% of childhood neoplasms and yolk sac tumor is the most common histopathological subtype [3]. Although, most germ cell tumors in children originate in the gonads, the most common primary site for YST is the sacrococcygeal region [4]. Due to the rarity of sacrococcygeal YST only few case reports and small series have been reported. 

Here we present a case of primary YST of the sacrococcygeal region.

## CASE REPORT

A 30-month-old infant presented for a right buttock soft tissue mass and constipation. The mass was a 6 cm x 4 cm nodular area of induration. The child had a normal birth history with no significant past medical history. Physical examination was otherwise normal for a healthy, appropriately developing baby. Serum alpha fetoprotein level was 706 ng/ml (normal range: 0-10 ng/ml). An ultrasound of the right buttock demonstrated a solid soft tissue hypoechoic mass, sized 6 cm x 4 cm. Abdomino-pelvic computerized tomography (CT) scan showed an irregular 6 x 7.5 x 5.5 cm solid mass of low-density invading the right greatest gluteal muscle. The mass was depressing the sigmoid and the rectum. No invasion of the spine was seen. Abdominal and pelvic magnetic resonance imaging (MRI) of the pelvis demonstrated a large heterogeneous pelvic mass extending into the right gluteal region with significant mass-effect on adjacent organs (Fig. 1). It showed infiltration of lower sacral spinal canal. No lesions were seen on a radiograph of the chest. 

**Figure:**
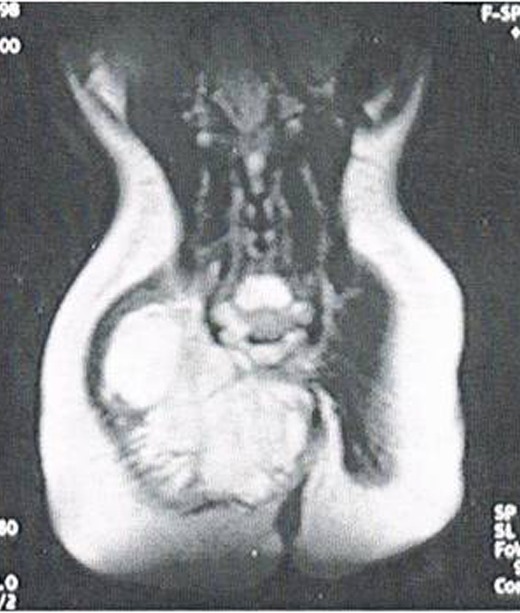
Figure 1: Abdominal and pelvic magnetic resonance imaging of the pelvis displays a large heterogeneous pelvic mass extending into the right gluteal region with significant mass-effect on adjacent organs. It shows infiltration of lower sacral spinal canal.

Surgical-biopsy consisted of whitish gray, friable and hemorrhagic fragments. Histopathological examination showed a tumor with papillary structures and several glomeruloid aggregates resembling Schiller-Duval bodies (Fig. 2). Neoplastic cells were relatively large, with pale eosinophilic to water-clear cytoplasm including some hyaline droplets with round to oval vesicular nuclei. Periodic acid-Schiff stain showed intracytoplasmic and extracellular eosinophilic globules, which were diastase resistant. Immunohisto-chemical stains for cytokeratin and AFP were strongly positive.

**Figure:**
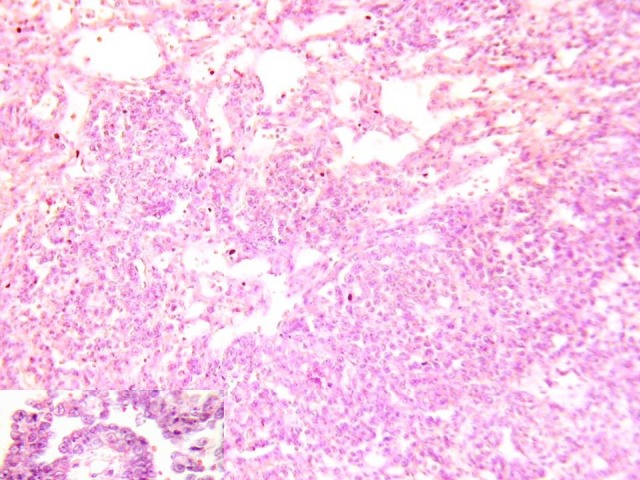
Figure 2: Histological features of a typical yolk sac tumor with lace-like (reticular) network of medium-sized cuboidal or flattened malignant cells and papillary structures (×40, H and E stain).

The patient completed five cycles of bleomycin, etoposide, and cisplatin neoadjuvant therapy. The AFP levels returned to normal by the start of the fourth course of chemotherapy. An abdomino-pelvic MRI performed at the completion of chemotherapy showed no residual tumor. The patient underwent a coccygectomy. The postoperative period was uneventful. After 3 years of follow-up, the patient remained without evidence of recurrent disease and serum AFP remained normal.

## DISCUSSION

According to Brown, germ cells in the developing embryo arise in the yolk sac, migrate around the hinder end of the primitive gut to the genital ridge on the posterior abdominal wall, and are finally absorbed into the developing gonads. It is suggested that during this migration, some germ cells may get left behind on the journey or may stray too far and come to rest at various sites along the dorsal wall of the embryo near the midline. The primordial germ cells give rise to an undifferentiated germ cell line. The undifferentiated germ cells undergo differentiation into embryonic (somatic cells) or extra –embryonic cells of yolk sac, chorion and allantoin cells. 

Patients with sacrococcygeal yolk sac tumor present most often with complaints of constipation or buttock swelling [4]. Sacrococcygeal YST develops exclusively in children less than 3 years of age [1, 4-6].

The imaging of sacral tumors in children has great values in identifying the position, contents and invasion. YST, consisting of fat-free soft tissue, is often complicated with hemorrhage, necrosis and cystic degeneration. Thus on CT and MRI the signal density of EST is heterogeneous. Honeycomb-like change is an imaging characteristic of YST [7]. Obscure boundary between tumor and surrounding tissue, sacral invasion and metastases are signs of malignancy. Each child with a sacrococcygeal germ cell tumor should be studied for alpha fetoprotein (AFP). AFP determination is useful in the diagnosis, to monitor the results of therapy and detects metastases and recurrence after therapy. The production and release of AFP is not limited to YSTs. It is commonly found, both in serum and in tissues, in embryonal carcinomas and teratocarcinomas containing only yolk sac elements and even in those without morphologically recognizable elements of this type [8]. However, AFP is also normally found in foetal serum and in that of the newborn infant [9]. Levels decrease and then reach adult levels about 8 months after birth [10].

Gross examination of YSTs typically reveals a mass that is predominantly solid and is soft, white, gray, or pale yellow. Cystic degeneration as well as necrosis and hemorrhage are often present.

Microscopic patterns of YST are numerous. They are described and illustrated by Teilum in his classical writings on this tumor [11]. Several different patterns are usually admixed. They are characterized by the intermingling of epithelial and mesenchymal elements in a specific organoid fashion. Micocytic, glandular-alveolar and papillary formations are common. Many of the cystic spaces are lined by flattened, endothelium-like layer of cells. The stroma can be quite cellular, spindle shaped, and reminiscent of smooth muscle. Perivascular Schiller-Duval bodies, which are almost always present in sizable samples but may be absent in limited material, as in biopsies, are the most distinctive features of yolk sac tumor. Periodic acid Schiff –positive hyaline intracytoplasmic and extracytoplasmic droplets are consistently seen in yolk sac tumors. 

Immunohistochemically, these tumors characteristically stain for alpha-fetoprotein. However, unlike serum levels which are rises in all cases of YST, immunohistochemical assay may be negative for AFP in some cases. Most of the immunoreactivity for AFP is not in the hotline globules but it is rather seen diffusely or in granular fashion throughout the cytoplasm of the tumor cells [8]. 

The treatment of malignant sacrococcygeal GCTs, such as primary yolk sac tumor is dependent on the extent of disease. Local disease is best managed surgically, while advanced tumor stages benefit best from adjuvant platinum based chemotherapy. Survival rates using these strategies are higher than 80% [12].

## Footnotes

**Source of Support:** Nil

**Conflict of Interest:** None declared
